# Identification and treatment of primary cervical gestational trophoblastic neoplasia: a retrospective study of 13 patients and literature review

**DOI:** 10.1186/s13023-021-02111-w

**Published:** 2021-11-18

**Authors:** Xiaoyu Wang, Junjun Yang, Xirun Wan, Fengzhi Feng, Jun Zhao, Tong Ren, Yang Xiang

**Affiliations:** Department of Obstetrics and Gynecology, Peking Union Medical College Hospital, Peking Union Medical College and Chinese Academy of Medical Science, National Clinical Research Center for Obstetric and Gynecologic Diseases, Shuaifuyuan No. 1, Dongcheng District, Beijing, 100730 China

**Keywords:** Cervical choriocarcinoma, Clinical features, Diagnosis, Gestational trophoblastic neoplasia, Treatment

## Abstract

**Background:**

Primary cervical gestational trophoblastic neoplasias (GTNs) are extremely rare ectopic GTNs. Such lesions are difficult to diagnose clinically because of their rarity, with abnormal vaginal bleeding of a non-specific cause being the most common symptom. To that end, this retrospective study aimed to identify the clinical characteristics of cervical GTN and to explore diagnostic and therapeutic strategies.

**Results:**

Thirteen patients diagnosed with primary cervical GTN at the Department of Gynecology, Peking Union Medical College Hospital, Beijing, China, between June 1, 1988 and May 31, 2020 were included in the study. All patients had irregular vaginal bleeding, including six who presented with massive bleeding. Seven patients (53.8%) were initially misdiagnosed with a cervical pregnancy. All patients received chemotherapy; 11 (84.6%) also underwent hysterectomy because of chemoresistant lesions or uncontrolled bleeding. All patients achieved complete remission; however, two women (15.4%) experienced a relapse during the median follow-up period of 35 months. A comprehensive review of English-language literature published between 1980 and 2020 identified 22 case reports encompassing 27 patients. The definitive diagnosis was achieved via pathology in 26 of them (96.3%), and hysterectomy was performed in 21 (77.8%).

**Conclusions:**

Owing to its rarity and nonspecific symptoms, the diagnosis of primary cervical GTN is challenging and often relies on pathology. The combination of chemotherapy and hysterectomy is the main therapeutic strategy for this disease.

## Background

Gestational trophoblastic neoplasias (GTNs) are a heterogeneous group of pregnancy-related disorders that include invasive mole, choriocarcinoma, placental site trophoblastic tumor (PSTT), and epithelioid trophoblastic tumor (ETT). GTNs usually originate in the uterine body and should be included in the differential diagnosis of patients with abnormal vaginal bleeding, symptoms of metastasis, and an elevated β-human chorionic gonadotropin (β-hCG) level.

Primary GTN lesions located outside the uterine body (referred to as ectopic GTNs) are very rare. Saito et al. [1] defined the diagnostic criteria for ectopic choriocarcinoma as having no primary choriocarcinoma focus in the uterine corpus, a pathological confirmation of choriocarcinoma as the type of lesion, and the ruling out of an extrauterine choriocarcinoma coexisting with a hydatidiform mole or of a normal intrauterine pregnancy with an intramural choriocarcinoma in the uterine corpus.

Primary cervical GTN is an extremely rare type of ectopic GTN that is difficult to clinically diagnose because of its rarity; non-specific abnormal vaginal bleeding is the most common symptom [2]. This disease can also mimic other, more common, cervical lesions such as cervical pregnancy, threatened abortion, cervical polyp, or cervical neoplasia. A missed or delayed diagnosis may result in severe complications either from the natural progression of the disease or from inappropriate treatment [3]. As such, we performed a retrospective analysis and literature review of the clinical characteristics and therapeutic processes of primary cervical GTN with the aim of contributing to improvements in the diagnostic accuracy of this disease and establishing better therapeutic strategies.

## Results

### Demographic data of the study population and preoperative diagnosis

Thirteen patients were included in this retrospective study (Table 1); their median age was 32 years (range 22–49 years). The median serum β-hCG level and uterine lesion size were 23,157 (range 255–157,324) mIU/mL and 4.5 (range 1.5–6.8) cm, respectively. Exophytic and endophytic lesions were found in four (30.8%) and nine (69.2%) of the patients, respectively. Eight patients had antecedent pregnancies that culminated in term delivery. The median International Federation of Gynecology Obstetrics (FIGO) score was 8 (range 3–11), and 15 patients (38.5%) had pulmonary metastases. The main clinical manifestation was irregular vaginal bleeding, which was reported in 12 patients (92.3%); six presented with massive vaginal bleeding and one underwent uterine artery embolization. Other presentations included amenorrhea (7.7%) and abdominal pain (15.4%). None of the patients reported pulmonary or neurological symptoms.

**Table 1:** Characteristics of patients

Characteristics	N (%), or median (range)
Age, year	32 (22–49)
Antecedent pregnancy	
Mole	1 (7.7%)
Abortion	4 (30.8%)
Term	8 (61.5%)
Interval since antecedent pregnancy, month	
Massive vaginal bleeding	6 (46.2%)
Pretreatment serum β-hCG, mIU/mL	23,157 (1023–239234)
< 10^3^	1 (7.7%)
10^3^–10^4^	4 (30.8%)
> 10^4^	8 (61.5%)
Lesion size, cm	4.5 (1.5–6.8)
FIGO stage	
I	8 (61.5%)
II	0
III	4 (30.8%)
IV	1 (7.7%)
FIGO score	
≤ 6	4 (30.8%)
> 7	9 (69.2%)

*FIGO* international federation of gynecology obstetrics

### Intervention and diagnosis

Nine of the patients (69.2%) were originally misdiagnosed: six (46.1%) with cervical pregnancy, two (15.4%) with cervical cancer, and one (7.7%) with incomplete abortion. The definitive diagnosis of GTN in all these nine patients was ultimately made following initial surgery, which involved curettage in five women (55.6%), cervical biopsy in two (22.2%), and hysterectomy in two (one for uncontrolled bleeding and the other for suspected cervical pregnancy; neither had childbearing plans). All the nine patients (69.2%) were found to have choriocarcinoma on pathological examination, while the remaining four (30.8%) were diagnosed clinically. Invasive mole and choriocarcinomas were detected clinically in one and three patients, respectively, and were confirmed by pathological examination after complementary surgery.

### Treatments and outcomes

All 13 patients received multiagent chemotherapy either immediately after completing their initial treatment or after undergoing pathological examination as an adjuvant treatment. The median number of chemotherapeutic courses was six (range 4–10). Complementary hysterectomy was performed on nine patients, four with tumor diameters exceeding 3 cm with no obvious shrinkage after regular multiagent chemotherapy, three with plateaued β-hCG levels, and two with no childbearing plans. Including two patients who underwent hysterectomy as initial surgery, hysterectomy was performed in a total of 11 (84.6%) patients. All patients achieved complete remission (CR) after the treatment. The median follow-up duration was 40 months (range 4–139 months); two women (15.4%) experienced recurrences in the cervix and lung, respectively, and underwent a combination of chemotherapy and surgery whereupon they re-achieved CR. Detailed information regarding these two patients is shown in Table 2.

**Table 2:** Detailed information about two women relapsed after initial treatment

Case (age)	Time since diagnosis, mo	β-hCG	Lesion location (size, cm)	FIGO stage	FIGO score	Initial surgery	Chemotherapy (number of courses)	Complementary surgery	Status (month since chemotherapy completion)
1 (22)	0	36,902	Cervix (4.6)	I	9	Curettage	EMA/CO (8)	–	Recurrence (4)
	8	8288	Cervix	I	11	–	FAEV (4)	Hysterectomy	CR (14)
2 (31)	0	2297	Cervix (3)	I	6	Curettage	FAV (2), EMA/CO (5)	Hysterectomy	Recurrence (4)
	4	83	Lung	III	7	–	FAEV (5)	Pulmonary lobectomy	CR (6)

*CR* complete remission; *EMA/CO* vincristine; and etoposide, methotrexate, dactinomycin/cyclophosphamide and vincristine, *FAEV* floxuridine, dactinomycin, etoposide, *FAV* floxuridine, dactinomycin, and vincristine, *FIGO* International Federation of Gynecology Obstetrics

### Literature review

Twenty-seven patients with primary cervical GTN were reported in 22 English-language publications over the past 40 years; their features are summarized in Table 3 [2–23]. The definitive diagnoses of these patients included 24 choriocarcinomas, two PSTTs, and one ETT; moreover, one patient was diagnosed during mid-term pregnancy. Our literature review found that 25 patients (92.6%) presented with irregular vaginal bleeding; 10 (37%) had massive vaginal bleeding.

**Table 3:** Cases of primary cervical GTN in a comprehensive literature review

Case	Reference	Age	Primary diagnosis	Primary treatment	β-hCG	Complementary surgery	Chemotherapy	Follow-up (month)
1	Fu et al. [2]	46	Abortion	Curettage	20,000	Hysterectomy	Yes	CR (17)
2		21	GTN	Chemotherapy	5344	Hysterectomy	Yes	CR (16)
3		35	Abortion	Curettage	4000	Hysterectomy	Yes	CR (14)
4		30	Abortion	Curettage	2764	Hysterectomy	Yes	CR (6)
5	Baykal et al. [3]	54	Cervical cancer	Cervical biopsy*, RH + BSO + BLN	45,000	No	Yes	NA
6	Ben et al. [4]	35	Cervical myoma	Curettage	NA	Hysterectomy	Yes	NA
7	Sorbi et al. [5]	30	CSP	Hysterectomy	60,000	No	Yes	NA
8	Lee et al. [6]	29	Cervical pregnancy	MTX + Hysterectomy	180,580	No	Yes	NA
9	Mitrovic et al. [7]	35	NA	Hysterectomy	13	No	No	NA
10	Herts et al. [8]	47	Cervical pregnancy	Hysterectomy	109,870	No	Yes	NA
11	Kairi-Vassilatou et al. [9]	43	Ectopic pregnancy	MTX + Curettage	7485	Hysterectomy	Yes	NA
12	Ben-Chetrit et al. [10]	33	Cervical cancer	Cervical biopsy*, Cervical conization	32	No	Yes	CR (12)
13	Frati et al. [11]	32	Cervical cancer	Cervical biopsy	30,750	No	Yes	NA
14	Yahata et al. [12]	38	Cervical polyp	Cervical biopsy	12,800	Hysterectomy	Yes	CR (14)
15	Chandacham et al. [13]	33	Cervical cancer	Cervical biopsy	45,724	No	Yes	CR (33)
16	Wang et al. [14]	27	NA	Cervical biopsy	1900	Hysterectomy	Yes	CR (8)
17	Tsai et al. [15]	46	GTN	Hysterectomy	162,550	No	Yes	NA
18		43	Cervical cancer	Hysterectomy	NA	No	Yes	NA
19		29	GTN	Hysterectomy	NA	No	Yes	NA
20	Park et al. [16]	31	NA	Cesarean section + tumor resection	NA	Hysterectomy	Yes	NA
21	Tsukamoto et al. [17]	42	Cervical cancer	Cervical biopsy*	8000	Hysterectomy	Yes	NA
22	Tripathi et al. [18]	28	NA	Curettage	54,000	Hysterectomy	Yes	CR (10)
23	Karaman et al. [19]	36	Ectopic pregnancy	MTX + Cervical biopsy	5374	No	Yes	NA
24	Phippen et al. [20]	40	Cervical cancer	Cervical biopsy* Cervical conization	20	Hysterectomy	No	NA
25	Wang et al. [21]	36	Cervical pregnancy	Cervical pregnancy	149,100	No	Yes	NA
26	Roopnarinesingh et al. [22]	25	Ectopic pregnancy	Laparoscopic exploration + cervical biopsy +curettage	4580	No	Yes	Relapse (6)
27	Zwischenberger et al. [23]	38	Cervical cancer	Cervical biopsy	13	Hysterectomy	No	NA

*BLN* bilateral pelvic-paraaortic lymph node dissection, *BS* bilateral salphingoopherectomy, *CR* complete remission, *CSP* cesarean section pregnancy, *GTN* gestational trophoblastic neoplasia, *MTX* methotrexate, *NA* not available, *RH* radical hysterectomy; *Pathology originally showed squamous cervical cancer

The primary diagnosis was incorrect or unclear for at least 20 of these patients: eight (29.6%) were misdiagnosed with cervical cancer, three (11.1%) with cervical pregnancy, three with ectopic pregnancy, three with abortion, one (3.7%) with a cervical myoma, one with cesarean section pregnancy and one with a cervical poly. Ten patients underwent a cervical biopsy; however, the samples obtained from four of them were originally interpreted as squamous cell carcinoma of the cervix on pathological examination.

A total of 21 (77.8%) patients underwent hysterectomy, including eight (29.6%) patients as their primary treatment and 13 during chemotherapy. Of the remaining six patients who wished to preserve their fertility, one experienced relapse in the cervix (identified by a large loop excision of the transformation zone) 6 months after chemotherapy. Long-term data were available for 10 patients after a median follow-up of 13 months (range 6–33 months). Except for the aforementioned patient who relapsed, the remaining nine patients had normal β-hCG levels at their last follow-up visit.

## Discussion

Our study encompassed 13 patients treated at the Peking Union Medical College Hospital (PUMCH) as well as 27 who were reported in the literature as having been diagnosed with primary cervical GTN. The uterine cervix is evidently an extremely rare location for the development of GTNs, the clinical presentations of which are nonspecific. Unlike most patients with GTN that can be diagnosed clinically and achieved CR by chemotherapy alone, the definitive diagnoses of primary cervical GTN were primarily made based on pathological examinations, while most patients underwent hysterectomy for uncontrolled bleeding or chemoresistant lesions. These findings highlight the importance of surgery both in the diagnosis of primary cervical GTN as well as in the treatment approach.

The pathogenesis of cervical GTN remains unclear. It may develop from a cervical metastasis arising from a primary tumor in the corpus that later spontaneously regresses; alternatively, it may constitute the malignant transformation of a cervically implanted fetus or else arise from chorionic cells from a preceding pregnancy that migrate to the site and undergo malignant transformation after a period of dormancy [4].

GTNs are solid tumors that can be diagnosed without histological evidence if patients present with typical clinical, laboratory, and radiographic features [24]; however, our retrospective study and literature review showed that patients with GTNs located in the uterine cervix had no specific clinical features. The majority of patients with this condition seek advice from physicians because of irregular vaginal bleeding, the rates of which among our patients and those identified in our literature review were 92.3% and 92.6%, respectively; these rates are higher than that associated with post-term choriocarcinoma (74%) [25]. Moreover, the incidence of uncontrolled massive vaginal bleeding was markedly high owing to the anatomical position of the lesion. Although cervical carcinoma, cervical myoma, and pregnancy-associated disorders may be more common manifestations of cervical masses associated with vaginal bleeding, significantly elevated serum β-HCG levels should alert clinicians to the possibility of a cervical GTN. Hence, given its importance as a marker, β-hCG should be monitored stringently.

**Fig. 1:**
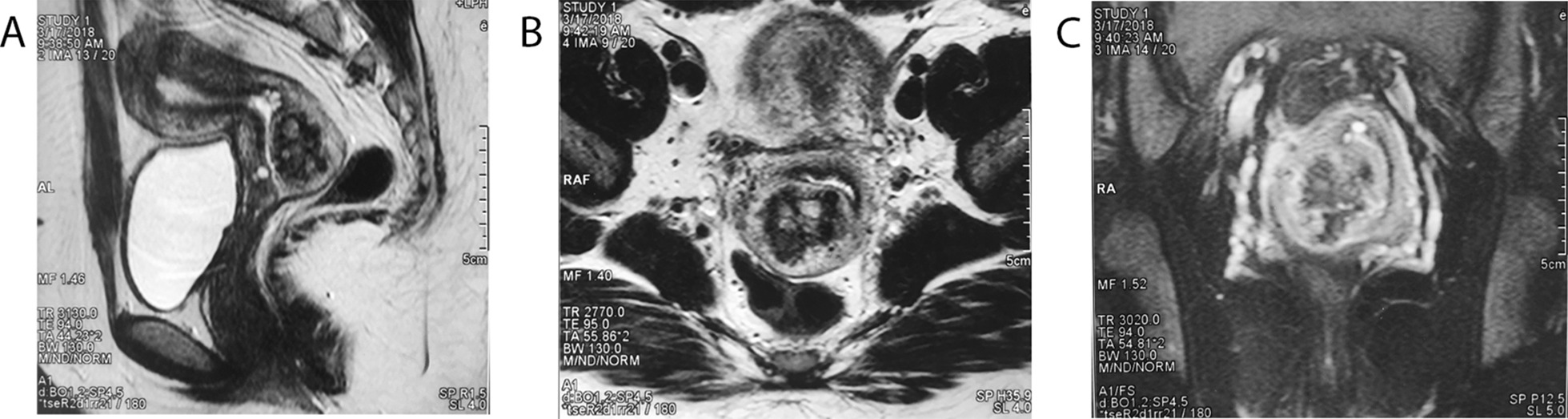
Magnetic resonance imaging of a 22-year- old woman with choriocarcinoma. Sagittal T2-weighted image (**a**) and axial T2-weighted image (**b**) demonstrate a heterogeneous, hyperintense, focal mass located in the posterior cervical lip. Coronal (**c**) contrast-enhanced T1-weighted images show heterogeneous enhancement of the tumor. Tortuous flow voids consistent with vessels can be observed in the parametrium, indicating tumor hypervascularity

Transvaginal color Doppler ultrasonography is essential for the early diagnosis of cervical GTN because it allows for the detection of hypervascularity (with diastolic blood flow) in tumoral vessels that arise owing to angiogenesis and neovascularization [12]. The typical vasculature has a turbulent appearance with color distortion, high velocity, and a low resistance index [26]; in comparison, a typical cervical pregnancy consists of a gestational sac in the mass. However, some advanced cervical cancers with hypervascularity are difficult to differentiate from GTN on ultrasonographic images, necessitating magnetic resonance imaging (MRI) to derive essential information regarding location, parametrial extension, and blood supply. On both T1-and T2-weighted images, abundant GTN vascularization exhibiting tortuous flow can be observed in various spaces. Hemorrhagic lesions appearing as areas of slightly higher signal intensity than the adjacent myometrium on T1-weighted images can best be observed using dynamic contrast-enhanced MRI (Fig. 1). Moreover, chest radiography and computed tomography may provide evidence of any pulmonary metastases.

While profuse bleeding may occur after biopsy, a histological diagnosis is important and may be lifesaving for patients with any of the aforementioned conditions. For those with cervical lesions that do not exhibit the typical manifestations described above, pathological examination should be considered for a definitive diagnosis. Notably, four patients in our literature review were misdiagnosed with cervical squamous cell carcinoma based on the original pathology, resulting in inappropriate treatment [3, 10, 17, 20]. Two of them were definitively diagnosed following cervical conization; the third after undergoing radical hysterectomy, bilateral salpingo-oophorectomy, and bilateral pelvic–paraaortic lymph node dissection; and the fourth following hysterectomy. The difficulty experienced by pathologists in distinguishing cervical squamous cell carcinoma (particularly the poorly differentiated type) from choriocarcinoma has been well described [3, 10, 17, 20]. Measuring serum β-hCG levels and reviewing pathologic slides subjected to immunocytochemistry are helpful for achieving the correct diagnosis, as evident in our findings.

Surgery for GTN excision can be very dangerous if bleeding is not controlled, especially considering the extremely high vascularization of these lesions. Uterine artery embolization is safe and effective for stopping excessive vaginal bleeding; this reduces the risk of hemorrhage before surgery and allows for successful conservative management using chemotherapy alone, thereby preserving fertility [11, 13]. Since successful pregnancies have been reported after uterine artery embolization in patients with GTN, such a procedure may be a safe and highly effective alternative in patients with cervical GTN, and ought to be the treatment of choice for women who wish to preserve their fertility. Hysterectomy may be considered in patients with uncontrolled uterine bleeding and in those who have no plans to become pregnant in the future [24].

Chemotherapy is considered as the primary treatment for GTNs, including in patients with distant metastases, and most patients could achieve CR by chemotherapy alone. A high risk FIGO score (> 6) and a clinicopathological diagnosis of choriocarcinoma are both associated with an increased risk of resistance to single-agent chemotherapy [24]; hence, multi-agent chemotherapy regimens are recommended for these patients. Only three patients in our literature review did not receive chemotherapy; their initial serum β-hCG levels were only 13–20 mlU/mL, and pathological examination revealed PSTT and ETT in two of them, respectively. Although surgery is considered less critical for the management of GTN, certain invasive procedures may be necessary to remove chemoresistant lesions in the uterus and metastatic sites as well as to control associated complications, especially for patients with PSTT and ETT. Hysterectomy was the initial treatment in 15.4% and 29.6% of patients in our retrospective study and among those we reviewed in the literature, respectively, and as many as 81.8% and 65%, respectively, underwent this surgical procedure during chemotherapy owing to the presence of chemoresistant lesions in the remaining patient. The high rate of surgery may be attributable to the large tumor size, which makes it difficult for the necrotic tissue to be absorbed; consequently, healing in this area may be relatively slow.

All patients in our retrospective study and literature review achieved CR following treatment. In all, 23 patients with cervical GTN underwent a prognostic analysis, among whom three relapsed 4–6 months after their last chemotherapy session. The recurrence rate was 13%, which was higher than that in a review of 1827 patients with GTN who achieved CR at our center, 118 (6.5%) of whom experienced recurrence during follow-up [27]. Notably, two of the three patients who relapsed in the study had not undergone hysterectomy, with the site of recurrence being the cervix. Thus, oncological safety should be considered when preserving fertility, especially in patients with a large tumor size.

One of the limitations of our study was its retrospective and observational nature; as such, the data were not randomized. Another limitation was that the sample sizes in both our study and in the literature were small; several patients were not included because they were reported in Japanese and other languages. Although a wider search was conducted for the years before 1980, chemotherapy was not widely used at that time and some patients died because they did not receive appropriate treatment. Longer-term follow-up periods are required to confirm our results.

## Conclusions

The early diagnosis of GTN located in the uterine cervix is challenging because of the rarity and nonspecific symptoms of this disease. GTN should be included in the differential diagnosis of cervical lesions in patients who are in their reproductive years, particularly when a cervical tumor is associated with profuse bleeding. Data from medical records, dynamic β-hCG monitoring, pelvic ultrasonography, MRI, and assessment of metastasis may contribute to the differential diagnosis, although histopathologic evidence is highly important in this regard. Chemotherapy is the preferred treatment, while hysterectomy is lifesaving when profuse cervical hemorrhage occurs and is also preventative of recurrence.

## Methods

### Study design and patient enrollment

Patients treated at the PUMCH in Beijing, China between June 1, 1988 and May 31, 2020 were included in the study. The patients were selected from the GTN database, and information on their clinical presentations, laboratory and imaging examinations, diagnostic and therapeutic procedures, and outcomes were collected from medical records. The inclusion criteria were as follows: (1) an apparent primary lesion located in the cervix based on visual examination, imaging features, and/or operative findings; (2) pathological diagnosis of an invasive mole, choriocarcinoma, PSTT, or ETT; and (3) regular treatment and follow-up. The exclusion criteria were (1) relapsed lesions located in the cervix, (2) non-gestational choriocarcinoma and (3) incomplete treatment or loss to follow-up.

### Intervention and diagnosis

Prior to treatment at PUMCH, all patients underwent disease evaluation including a complete review of their medical history; blood testing including routine blood examinations, evaluation of hepatorenal function, and determination of the serum β-hCG level; and pelvic ultrasonography and/or MRI. Distant organs were assessed for metastases when a GTN was suspected. An invasive mole was diagnosed when the lesion invaded the myometrium and an insufficient decrease in β-hCG levels after curettage of a molar pregnancy was observed. In patients after a nonmolar pregnancy, GTN was diagnosed based on an extremely high β-hCG level, radiographic features of the lesion(s), and/or the presence of distant metastases. In patients lacking all of these representative features, GTN was diagnosed by pathological examination. All pathologic analyses were conducted by pathologists with more than 10 years of diagnosis experience. The disease was staged and scored based on the FIGO 2000 risk factor scoring system.

### Chemotherapy and surgery

Chemotherapy was administered as the primary treatment when the diagnosis of GTN was definitive. A single-agent chemotherapy regimen, such as dactinomycin or methotrexate, was administered to low-risk patients (FIGO score 4 or lower). Since higher score and clinicopathologic diagnosis of choriocarcinoma are associated with an increased risk of resistance to single agent chemotherapy [24], other patients received multidrug chemotherapy, including floxuridine, dactinomycin, and vincristine (FAV); floxuridine, dactinomycin, etoposide, and vincristine (FAEV); and etoposide, methotrexate, dactinomycin/cyclophosphamide and vincristine (EMA/CO). Any chemotherapy-related toxicity as well as serum β-hCG levels were examined weekly, and pelvic ultrasonography was performed every two or three courses.

Some patients underwent uterine artery embolization owing to massive vaginal bleeding; moreover, hysterectomy was performed in patients with uncontrolled vaginal bleeding, those suspected of having a cervical pregnancy, those who had no childbearing plans, those whose serum β-hCG level didn’t declined logarithmically or as plateaued, and those whose tumor diameters exceeded 3 cm with no obvious shrinkage after regular multiagent chemotherapy. Three cycles of consolidation chemotherapy were administered after surgery.

### Follow-up

After treatment completion, all patients underwent regular and long-term follow-up for serum β-hCG. CR was considered achieved when the serum β-hCG level returned to normal and remained as such for at least four consecutive weeks, whereas relapse was defined as an elevated serum β-hCG level 3 months after complete remission.

### Review of the literature

A comprehensive review of literature published between January 1980 and December 2020 was conducted using the PubMed, MEDLINE, Embase, and Scopus databases; the primary objective was to identify all documented patients with primary cervical GTN in the English-language publications. The following keywords were used: (cervical OR cervix) AND (choriocarcinoma OR invasive mole OR placental site trophoblastic tumor OR epithelioid trophoblastic tumor). The inclusion criteria were retrospective studies, case series, and case reports of primary cervical GTN; exclusion criteria were articles in languages other than English, reviews, conference abstracts, non-gestational choriocarcinoma, and manuscripts with unavailable full texts. References of the identified articles were also screened for relevant publications. The following data were extracted from the articles included in the literature review: author and year of publication, number of reported patients; each subject’s age, symptoms, β-hCG level, tumor size, tumor location, initial diagnosis, method of definite diagnosis, histopathological features, and treatment modalities and outcomes.

### Statistical analysis

The results are presented as medians with interquartile ranges for continuous data, and as numbers with percentages for categorical variables. Data analysis was performed using SPSS version 22 (IBM Corp., Armonk, NY, USA).

## Data Availability

The datasets used and analyzed during the current study are available from the corresponding author on reasonable request.

## Abbreviations

β-hCG: β-human chorionic gonadotropin
CR: Complete remission
ETT: Epithelioid trophoblastic tumor
FIGO: International federation of gynecology obstetrics
GTN: Gestational trophoblastic neoplasia
MRI: Magnetic resonance imaging
PSTT: Placental site trophoblastic tumor
PUMCH: Peking Union Medical College Hospital
